# Primary Hyperparathyroidism From Concurrent Parathyroid Hyperplasia and Ectopic Parathyroid Adenoma: A Case Report

**DOI:** 10.1155/crie/2316653

**Published:** 2026-01-06

**Authors:** June Yao, Clarice Szeto, Chantal Riba, Chau Nguyen

**Affiliations:** ^1^ Department of Surgery, Community Memorial Hospital, 147 N. Brent Street, Ventura, 93003, California, USA, cmhshealth.org

**Keywords:** case report, ectopic parathyroid adenoma, intraoperative PTH, parathyroid hyperplasia, parathyroidectomy

## Abstract

Primary hyperparathyroidism (PHPT), whether caused by an adenoma or hyperplasia, can be curative with parathyroidectomy. However, persistently elevated parathyroid hormone (PTH) despite parathyroidectomy suggests multigland disease. We present a case of concurrent single‐gland parathyroid hyperplasia and an ectopic parathyroid adenoma in a 72‐year‐old woman with long‐standing PHPT. Despite persistently elevated calcium and PTH levels, all diagnostic imaging was negative for parathyroid hyperplasia and adenoma. PTH remained elevated despite initial parathyroidectomy for hypercellular tissue consistent with hyperplasia. An ectopic paraesophageal parathyroid adenoma was ultimately discovered and resected, resolving the PHPT. This case emphasizes that negative imaging does not exclude active parathyroid disease.

## 1. Introduction

Primary hyperparathyroidism (PHPT) is characterized by excessive secretion of parathyroid hormone (PTH) and is the most common cause of hypercalcemia. Parathyroid adenoma accounts for 80%–85% of PHPT cases, while parathyroid hyperplasia represents approximately 15%–20% [1]. The average parathyroid gland is approximately 6 mm by 4 mm, weighing only 20–40 mg [2]. Removal of the affected glands is usually curative. Persistent or recurrent PHPT can be attributed to inadequate resection secondary to an ectopic gland (estimated at up to 5%) or double adenoma, which may be found in 3%–12% of the time [3].

Ectopic parathyroid glands result from abnormal migration of parathyroid cells during embryogenesis. Inferior parathyroids are most frequently associated with the thymus or the thyroid gland, while the most common positions for ectopic superior parathyroids are the tracheoesophageal groove and retroesophageal region [4]. Ultrasound and sestamibi scintigraphy remain the primary localization studies for enlarged parathyroid glands. When initial imaging is inconclusive, contrast‐enhanced CT, MRI, or advanced studies such as 4D‐CT, PET radiotracers, or intraoperative gamma probes may be indicated [5, 6]. Upon literature review, only one similar case of coexisting parathyroid hyperplasia and ectopic parathyroid adenoma has been reported. In this case report, we present a rare case of PHPT due to single‐gland parathyroid hyperplasia and an ectopic paraesophageal adenoma, managed successfully with bilateral neck exploration and intraoperative PTH monitoring. This case report has been reported in line with the CARE Criteria [7].

## 2. Case Presentation

A 72‐year‐old Caucasian female with a long‐standing history of PHPT presented with recurrent hospital admissions for symptomatic hypercalcemia. She had an extensive medical history that included hypertension, hyperlipidemia, Type 2 diabetes, heart failure with preserved ejection fraction, bipolar disorder, uterine cancer with metastases to the lung treated with total abdominal hysterectomy, and left lower lobectomy with adjuvant chemotherapy and radiation.

She had two hospitalizations in the prior 2 months for progressively worsening encephalopathy. Laboratory data during these admissions showed PTH level of 311 pg/mL (normal range 12–88 pg/mL) and uptrending hypercalcemia of 14.5 mg/dL (normal range 8.6–10.2 mg/dL). CT abdomen and pelvis demonstrated an obstructive calculus of the left kidney. She did not have any kidney disease prior to this episode. She was treated with cinacalcet, calcitonin, as well as a nephrostomy tube placement before surgical consultation.

On examination, the patient’s neck was noted to be large and short without any palpable masses or lymphadenopathy. She was easily arousable; however, she was confused and was only oriented to person and place. Laboratory data on admission revealed a corrected serum calcium markedly elevated at 17.3 mg/dL. Her albumin was 3.2 g/dL (3.5–4.8 g/dL).

## 3. Diagnostic Assessment

Diagnostic imaging of CT neck, neck ultrasound, and sestamibi scan completed on hospital Day 4 did not reveal evidence of parathyroid adenoma. CT chest was also negative for a mediastinal mass that could have suggested an intrathoracic mediastinal ectopic parathyroid adenoma. On a personal review of the CT scan by the surgeon, a right peri‐thyroid cystic nodule was found on imaging and felt to be a potential adenoma. With her PTH level more than doubled at 700 pg/mL on hospital Day 9, the patient was taken for a bilateral neck exploration.

## 4. Therapeutic Intervention

The right neck was explored first. The aforementioned nodule was found just overlying the right recurrent laryngeal nerve but was shown to be benign thyroid tissue. The left neck was then explored, and an enlarged superior gland was found, measuring 1.8 × 1.1 × 0.6 cm and weighing 0.61 g. Pathology revealed hypercellular parathyroid tissue. Unfortunately, pictures were not taken. Fifteen minutes post‐excision, PTH level was still elevated at 685 pg/mL. Further exploration of bilateral neck and Level VI lymph nodes did not reveal any apparent adenomas. It was decided to terminate the surgery after a thorough exploration. The other parathyroid glands were not biopsied given their normal appearances. Given the patient’s comorbidities and altered mental status prior to surgery, the patient remained intubated and transferred to the intensive care unit (ICU).

**Figure 1:**
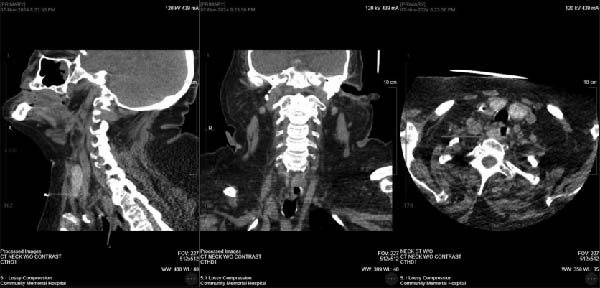
Right paraesophageal mass shown on CT scan.

**Figure 2:**
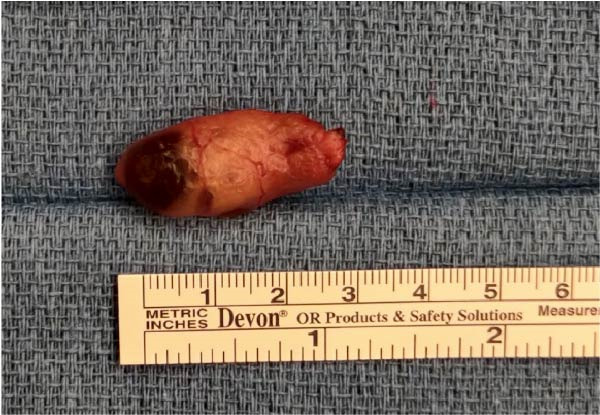
Gross image of right paraesophageal parathyroid adenoma.

Over the course of a week, the patient’s PTH rose to a similar preoperative value of 758 pg/mL. Upon further surgeon review of CT scan, a right paraesophageal mass was identified at the level of the clavicle, displacing the esophagus to the left, as shown in Figure 1. The patient was then taken back for a re‐exploration. The right thyroid gland was medialized to expose the trachea, and the dissection was taken down to the level of the clavicle and tracheoesophageal groove. An enlarged cystic mass was identified, shown in Figure 2. It measured 3.8 × 1 × 0.7 cm and weighed 2.61 g. Fifteen minutes intraoperative, PTH level dropped to 42 pg/mL from 758 pg/mL preoperatively. The patient remained intubated and was transferred back to ICU in stable condition.

## 5. Follow‐up and Outcomes

The patient had a slow recovery postoperatively. She was extubated on postoperative Day 4. Her encephalopathy improved but was ongoing due to other comorbidities. With her chronic hyperparathyroidism, there were concerns of hungry bone syndrome. Serum calcium downtrended from 9.1 mg/dL POD1 to 7.7 mg/dL POD7, repleted parenterally and orally. PTH remained within normal limits after surgery.

## 6. Discussion

This case report presents a diagnostically complex instance of coexisting parathyroid hyperplasia and ectopic parathyroid adenoma. Initial left superior parathyroidectomy pathology indicated hypercellular parathyroid tissue, consistent with hyperplasia. Two putative inferior parathyroid glands appeared grossly normal and were not biopsied, making four‐gland hyperplasia less likely but not definitely excluded. Persistent elevation of PTH prompted a detailed surgeon‐led re‐evaluation review of imaging with the radiologist, which identified a right paraesophageal mass that was subsequently resected during re‐exploration. Pathology confirmed a right parathyroid adenoma, and its removal resulted in the resolution of refractory PHPT.

Ultrasound and sestamibi scintigraphy are the primary preoperative imagings to identify and localize parathyroid adenomas, with reported sensitivities of 72%–89% and 68%–98%, respectively [8, 9]. Localization accuracy sensitivity improves to 96% when both studies are performed [10], reducing the need for bilateral neck exploration. However, the sensitivity declines when evaluating for multiglandular disease, as demonstrated in our case, creating a new diagnostic challenge [11].

After surgery, persistent and recurrent disease is most commonly seen in patients with double parathyroid adenomas, which occurs in 3%–12% of patients with hyperparathyroidism [3]. There is ongoing debate as to whether double adenomas represent a spectrum of parathyroid hyperplasia. In our patient, the enlarged left superior gland and the right superior ectopic adenoma were morphologically distinct—the latter consistent with a typical encapsulated tumor and the former broader and more diffuse. However, it is worth noting that a definitive pathologic distinction between hyperplasia and adenoma can be challenging with full glandular sampling, which is a limitation in this case.

In the past 50 years, only one similar case of coexisting parathyroid hyperplasia and ectopic parathyroid adenoma has been reported by Xie and Huang [8]. A ^99 m^Tcsestamibi scan identified a left superior hyperplastic parathyroid gland, but persistent PTH elevation led to a later discovery of a retrosternal ectopic parathyroid adenoma on repeat sestamibi scan [8]. This case further underscores the known limitations of sestamibi, ultrasound, and CT imaging in detecting multigland or ectopic disease.

Our case differs from the previously reported instance of single‐gland hyperplasia with ectopic adenoma in that all first‐line imaging—sestamibi, ultrasound, and CT—failed to localize the pathology, despite the patient presenting with severe symptomatic hypercalcemia and markedly elevated PTH levels. This case report highlights the limitations of standard imaging in detecting ectopic or multigland disease and emphasizes the importance of detailed review when clinical suspicion remains high. In complex scenarios such as this, bilateral cervical exploration coupled with intraoperative PTH monitoring remains a valuable approach to reduce the risk of missed pathology and re‐operation.

## Consent

The authors confirmed that written informed consent for publication of the case details and accompanying images was obtained from the patient’s daughter.

## Disclosure

All authors reviewed and approved the final draft.

## Conflicts of Interest

The authors declare no conflicts of interest.

## Author Contributions

June Yao, Chantal Riba, and Chau Nguyen were involved in the diagnosis and management of the patient. June Yao, Clarice Szeto, and Chantal Riba were involved in data collection. All authors were involved in writing and editing the manuscript.

## Funding

The authors received no specific funding for this work.

## Data Availability

The data that support the findings of this study are available from the corresponding author upon reasonable request.
